# Lower incisor position in different malocclusions and facial patterns

**DOI:** 10.4317/medoral.18434

**Published:** 2012-12-10

**Authors:** Estrella Hernández-Sayago, Eduardo Espinar-Escalona, Jose M. Barrera-Mora, Maria B. Ruiz-Navarro, Jose M. Llamas-Carreras, Enrique Solano-Reina

**Affiliations:** 1 Master of Orthodontics and Dentofacial Orthopedics. University of Seville; 2 Associate Professor of Orthodontics. School of Dentistry. University of Seville; 3 Chairman of Orthodontics. Stomatology Department. University of Seville. Seville, Spain

## Abstract

Introduction: The position of lower incisor has been of considerable concern when planning an orthodontic treatment, having been recognized as one of diagnostic keys, Very important in the development of malocclusion and facial pattern. Objectives: In this study we claim to determine the importance of the position and inclination of lower incisor in the different malocclusions and facial patterns, and to base which of the cephalometric measurement parameters are the mostreliable. Material and Methods: Ninety lateral radiographies were taken, and they were classified by skeletal malocclusion and facial pattern.These teleradiographies have been performed cephalometric analysis, which includelower incisor position belong the following analysis: Ricketts, Riolo, Tweed, McHorris, Jarabak-MSE and Holdaway. Study Design: Cross-sectional study where we perform statistical analysis Anova test, Pearson correlations and Bonferroni analysis. Results: The analyzed measurements present a statistically significant differentiation in lower incisor inclination respect to the anterior cranial base, McHorris angle, angulation of lower incisor respect to occlusal plane and mandibular plane. Conclusions: There are statistically significant differentiation in lower incisor position and inclination respect the malocclusion and individual facial pattern.

** Key words:**Lower incisor, cephalometric analysis, facial patterns, facial biotype, skeletal malocclusions.

## Introduction

Lower incisor and its position in the lower arch is considered to be of prime importance at time of planning an orthodontic treatment, having been this recognized as one of the keys in the orthodontic diagnostic.This paper of crucial importance in orthodontics is giving by its effects on aesthetic and stability (1).

Relation between function and shape, such as it is described in the evolutionist principles, can be applied to orthodontic patients through skeletal compensations and, more evidently, through dentoalveolar compensations since nature need to have, in order to compensate, basic pathology present in the genetic code of the individual (2).

The role of dentoalveolar compensation in the development of a normal occlusion has been described in many articles (3-5). Similarly, the incisal adaptation to changes that sagittal maxillo-mandibular relationship suffers during growing, has been demonstrated in many longitudinal studies (6).

With these ideas in mind, it can be stated there is a close relationship between the antero-posterior relationship of the jaw or mandible, and the incisor inclination (7,8). So, lower proinclined incisor is associated to a delayed position of the jaw. Besides, retroinclined incisor is found in skeletal patterns of the mandible in forward position (9). This is known as a mechanism of den-toalveolar compensation (10).

Quantitative evaluation of adjustment of the dentoalveolar process, as a compensatory mechanism of sagittal malocclusion, may provide additional information on the orthodontic treatment to undertake (11).

Thus, skeletal malocclusions, frequent deformities in our society, can be addressed by two therapeutic routes, the orthodontic treatment, counterbalance of the problem, or the option of orthognathic surgery, combined with orthodontic treatment (12).

In a surgical option, it is a frequent objective of the pre-surgical orthodontic, to decompensate the inclination of a lower incisor, which it can be hidden or at least, doing less evident the basic skeletal pathology. As a result, it makes easier the obtainment of more favourable post-surgical results (13). In contrast, in the option purely orthodontic, the followed objective is to compensate even more, if it proceeds, the inclination of the lower incisor; i.e. lingual in Class III, and vestibular in Class II (14,15).

Many investigators (16,17) have examined the alveolar bone morphology in the lower incisive area. The labiolingual inclination, of the lower incisor, keeps a close and direct relationship with the labiolingual inclination of the alveolar bone, in the incisor area. Findings point out when the lower incisor is retroclined then, the alveolar bone also it is. Therefore, shape of the alveolar bone, in the incisor region, corresponds with the inclination of the lower incisor (4,15).

However, labiolingual inclination of lower incisor, not only it is associated to the inclination of the alveolar bone of the zone, but also to bone thickness. In this manner, front anretroinclined incisor, the alveolar bone of the region is showed more narrow, characterized by a lesser extend of the incisor root apex to external cortex of the labial face of the alveolar bone. As a consequence, it must be put special attention to movement, of this piece, at the moment of orthodontic treatment. This narrow alveolar gets even stronger in patients with dolicofacial patterns (16,17).

Along orthodontics history, many cephalometric methods have been proposed to determine the most suitable and stable position of lower incisor in the mandibular symphysis, to achieve satisfactory results. In our study, we pretend to determine which of these methods that contemplate incisive position may be more reliable in establishing the best position of the lower incisor, given the importance it has, and indicating those cephalometric parameters most statistically significant.

Most of the published articles only describes a few incisor position measures. We compared nine of the most commonly used methods to find the most significant to establish the correct lower incisor position.

In addition, we try to relate those incisive positions in orthodontically untreated subjects with different skeletal maloclussion and facial patterns. We hypothesized that the lower incisor natural position will be different depending on the pattern of each individual face, and it would be desirable the individualization of standards and clinical cephalometric guidelines for patients with neutral, vertical or horizontal growth.

We used lateral cephalograms because it had been considered the best method for diagnosis the lower incisor position before the 3D-tomography apparition. But, for our study, we don’t consider justified to bring under the subject to a higher radiation dose, being enough the information obtained for the 2D conventional radiography.

## Material and Methods

Sample is constituted by ninety patients divided in equal groups, all in function of their rated skeletal malocclusion, and valued through “Wits” appraisal, (male clinical standars-1±2, and female 0±2. Higher values are significant of Class II malocclusions, while values below the standard indicate Class III(18). At the same time, these groups were classified according to the facial pattern of the individual, who were analyzed through Jaraback growth spheres, defined as the percentage ratio between the posterior facial height (Se-Go)/anterior facial height (Na-Me) x 100. When this percentage oscillated from 54 to 58%, it indicates vertical growth. Higher values to 64% involve counterclockwise growth. Percentages from 59 to 63% correspond to a neutral growth.

**Figure 1:**
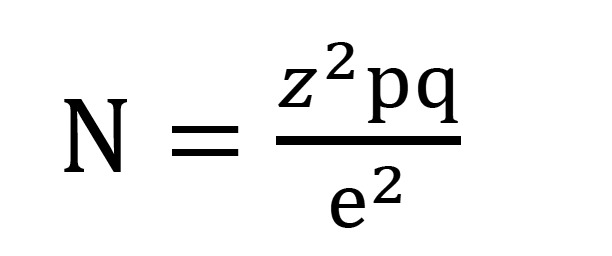
Formula to calculate the sample size.

We realized a pilot study on 20 patients to determine the sample size according to the following formula (Fig. 1):

-N: is the sample size.

-z: is the value corresponding to a confidence level, estimated at 95% (z= 1.96).

-pq: population variance. It’s estimated in the pilot study in 0,510.

-e: desired level of precision. We establish it in 5%.

According to this pilot study, we determined that for a standard error of 5% requires a sample size of 78 patients.

Previous studies (19-21) on the subject matter used similar total samples of patients. Based on this, and after having made a sample size analysis to determine the minimum sample size for this to be statistically significant, in our study we taken ninety patients, divided according to skeletal malocclusion attending the Wits appraisal and facial pattern. Based on these analyzes, we obtain a total sample of 90 subjects who were divided into homogeneous groups in response to skeletal Class and their facial pattern.

This sample was applied different cephalometric analyzes which provide the position and inclination of the lower incisor, per-forming therefore linear and angular measurements, as shown in ( Table 1).

**Table 1:**
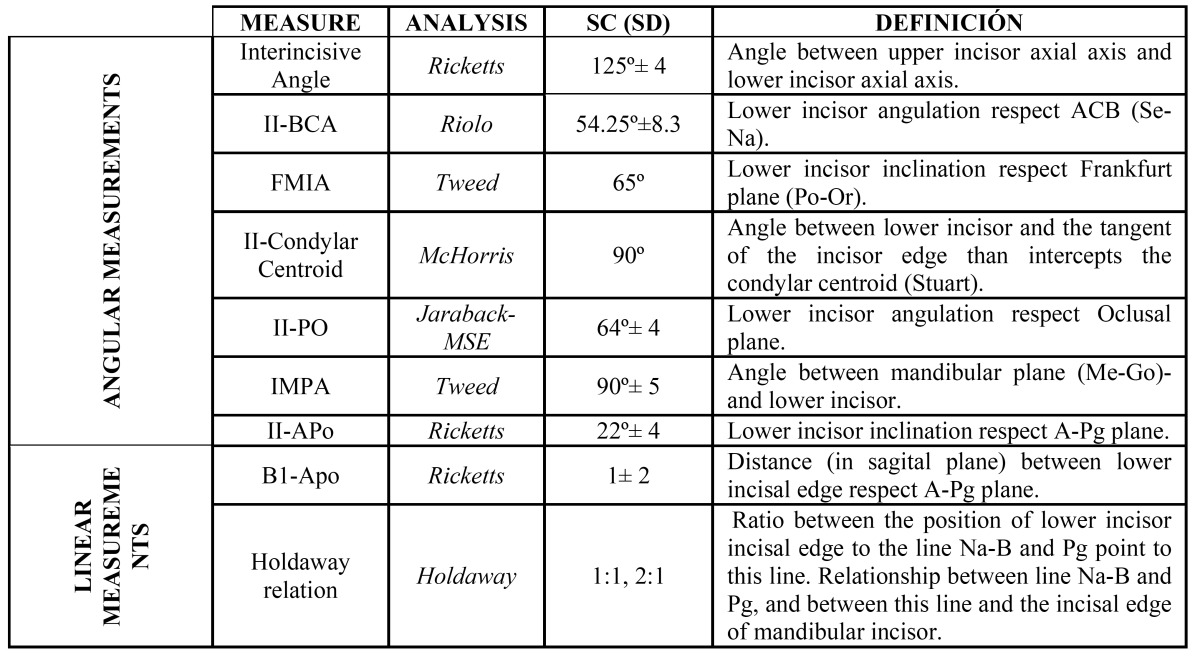
Measurements used in the study.

Angular Measurements:

-Interincisive angle (Ricketts analysis)

-Lower incisor respect to anterior cranial base (Riolo analysis)

-Inclination of lower incisor respect to Frankfurt plane (Tweed analysis)

-Inclination of lower incisor respect to condylar centroid (McHorris analysis)

-Angulation of lower incisor with the functional occlusal plane (Jarabak-MSE analysis)

-Inclination of the incisor respect to the mandibular plane (Tweed analysis)

-Angulation of the incisor respect to the dentation plane of Ricketts, A-Pg (Ricketts analysis)

Linear Measurements:

-Distance in the sagittal plane, of the incisal border of the inferior incisor, respect to the dentation plane of Ricketts, A-Pg (analysis of Ricketts)

-Proportion between the position of the incisal edge of the inferior incisor, respect to the Nasion - B line, and the pogonion point in the same line (analysis of Holdaway).

**Figure 2:**
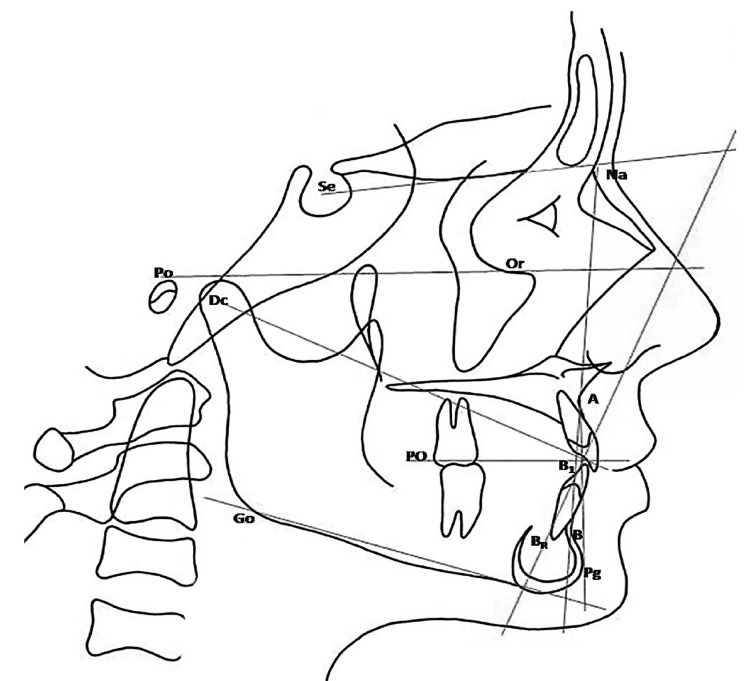
Cephalometric landmarks and measurements used.

In figure 2 it shows the measurements employed.

We use NemoCeph Dental Studio® NX 2006, version 6.0, Nemotec® program to obtain angular and linear measurements in different cephalometric analysis made; and compare the results obtained with those corresponding norms for such measurements.

Once obtained all data, they must be introduced into the statistic program, SPSS® version 15.0, so they can be analyzed very quietly and, therefore, obtain tables and correlation graphics. The Anova tests, Bonferroni test as well as the Pearson correlation analysis were employed.

The followings exclusion criterions have been used in this study:

-Patients younger than 16 years old, because we analyzed the lower incisor position in an adult and young adult sample.

-Patients that show a negative or positive discrepancy bone-dental that exceeds 3 mm.

-Patients who have been treated orthodontically.

-Patients who presenting discrepancy between skeletal malocclusion and the dentition malocclusion.

-Patients whose facial patterns differ in function of the analysis type: Vert of Ricketts versus Jaraback areas.

## Results

The analyzed parameters, according to the Anova test, show values statistically significant when analyzing differences found in different skeletal malocclusions which are: the angulation of the lower incisor respect to the anterior cranial base, the McHorris angle, and the angulation of the inferior lower respect to mandibular plane and to occlusal plane; all of them with a signification of p=0.000, except McHorris angle, with a data of p=0.002 ( Table 2).

**Table 2:**
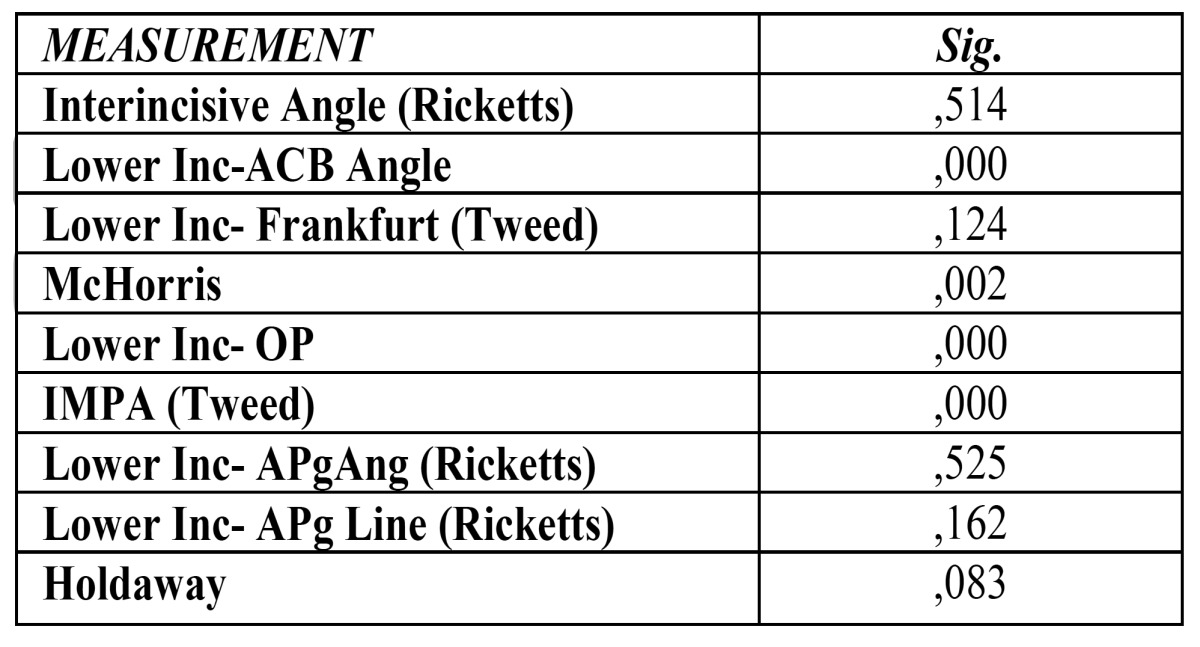
Results for ANOVA test for skeletal malocclusions.

According to Bonferroni analysis, realized with more details than the anterior, it can be observed how the encountered differences are statistically significant in the angulation of the inferior incisor respect to the anterior cranial base between skeletal malocclusions of Class I and II, and Class II and III. In McHorris analysis we obtained differences statistically significant between Class II and III. When we studied the angulations of the inferior lower respect to occlusal plane, we found differences statistically significant between all the types of skeletal malocclusions (Class I and II, Class I and III and Class II and III). Same results occur in the analysis of lower incisor respect mandibular plane.

When the incisor positions are interrelated with facials patterns, it shows a statistical significance in the angulation of the lower incisor respect to ACB (p=.000), respect to Frankfurt plane (p=.008), as well as position and angulation of lower incisor respect A-Pg plane (Ricketts) (p=.024 and p=.003, respectively) ( Table 3).

**Table 3:**
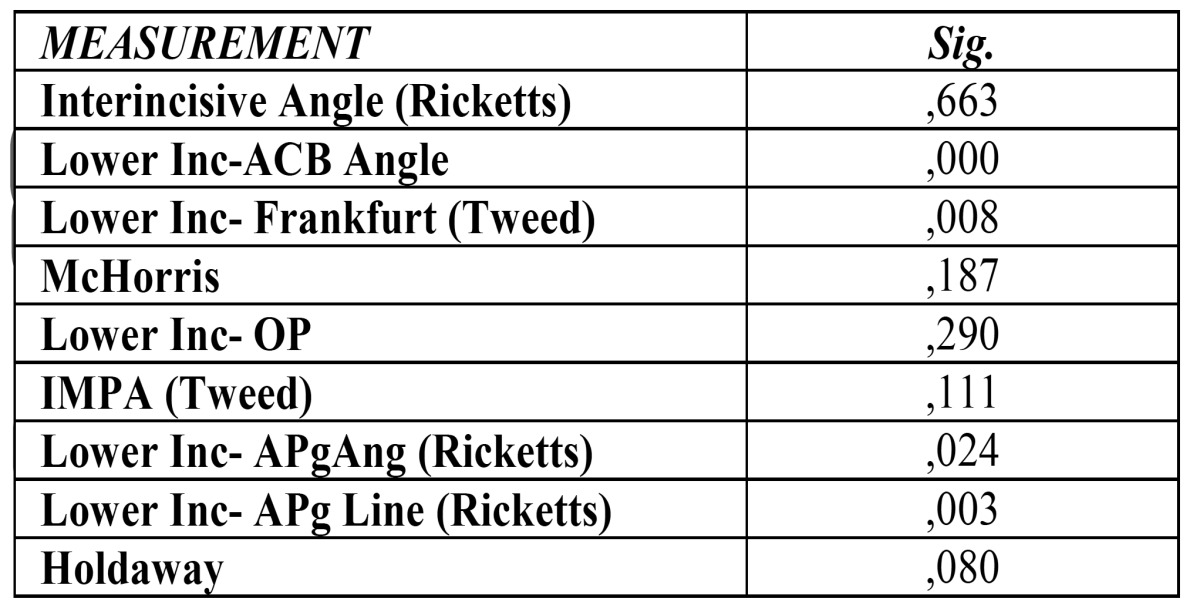
Results for ANOVA test for facial patterns.

Applying the Bonferroni analysis, we can prove that there are statistically significant differences in angulation respect to ACB between braqui and dolicofacial patterns. As to the angulation with Frankfurt Plane, between meso and dolico patterns and between braqui and dolicofacial. In Rickets analysis, it results statistically significant both the lower incisor position and inclination respect A-Pg plane between braqui and dolico patterns.

To analyze the relationship between skeletal malocclusion and facial pattern is performed univariate analysis of variance using Bonferroni analysis. It applies to the different groups, divided into 9 separate subgroups according to the type and facial pattern, resulting in statistically significant:

-Lower incisor angulation respect ACB (in Class II dolicofacialwith a statistically significant lower mean value with all other groups; and between Class III meso and braqui with a statistically significant higher mean value)

-IMPA (Class III dolico with a statistically significant lower mean value with the other malocclusion and facial patterns; as well as Class II dolicowith a statistically significant higher mean value with all Class III patterns, and with the Class I and II mesofacial; also Class II braquiwith a statisticallysignificant lower mean value with all the groups except Class II meso and dolico).

-Lower Incisor angulation respect Frankfurt plane (in Class II dolicowith a statisticallysignificant lower mean value with all other groups, excluding Class II braquifacial).

-McHorris analysis (Class II dolicofacial with a statistically significant lower mean value with Class I meso and braqui, Class II meso and Class III dolico and braqui).

-Lower Incisor- OP (Class II dolico with a statistically significant lower mean value with all the Class III patterns and with Class I braqui; and between Class III dolico with a statistically significant higher mean value with all the Class II patterns and Class I meso and dolico).

-Lower Incisor position - APg Ricketts (in Class I braqui with a statistically significant lower mean value respect Class II meso and dolico; and Class III dolico and braqui).

**Figure 3:**
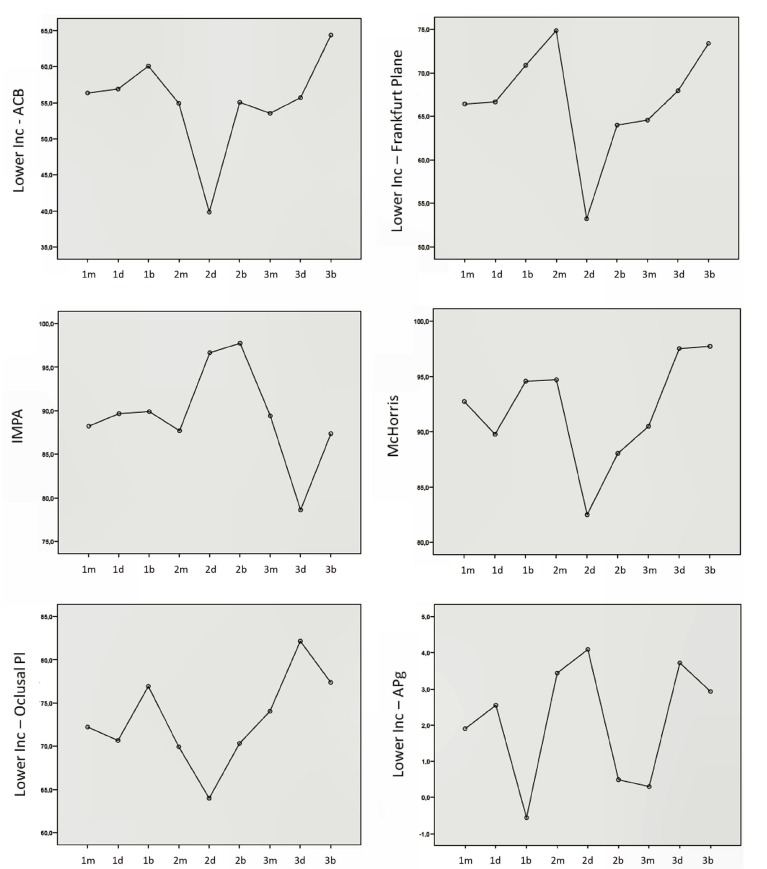
Graphics of the means statistically significant measures (Lower incisor-ACB, lower inc-Frankfurt PL, IMPA, McHorris, lower inc-Oclusal Pl, Lower inc position-APg.Group 1m: Class I mesofacial. Group 1d: Class I dolico. Group 1b: Class I braqui. Group 2m: Class II meso. Group 2d: Class II dolico. Group 2b: Class II braqui. Group 3m: Class III meso. Group 3d: Class III dolico. Group 3b: Class III braqui.

Graphics of statistically significant analysis, are showed at continuation: (Fig. 3).

Analysis of Pearson pretended to prove or confirm the interrelation between different analysis used in the position and inclination study of the inferior incisor. Thus, it was demonstrated that there is an intense and statistically significant relationship between the cephalometric analyses, except the interincisive angle and Holdaway analysis.

Therefore, it was found a directly proportional relationship among the inclination of the lower incisor respect to the anterior cranial base, McHorris analysis, lower incisor inclination respect Frankfurt plane and to the occlusal plane, respectively.

Nevertheless, it was established a relationship also statistically significant, but inversely proportional in this case between the lower incisor inclination respect to the mandibular plane, and the analysis explained above, as well as between these results and the Ricketts analysis, both position and inclination.

Ricketts analysis, respect to the position and inclination of the inferior incisor, keep a relation statically significant and directly proportional between them and the inclination of the incisive respect to the mandible plane, and inversely proportional with the rest of the commented analysis.

## Discussion

Dentition would be analyzed as equilibrium between dentoalveolar processes and the surrounding musculature, because the natural oral function has a relation with the right position of the inferior incisor within a facial harmony, considering dentoalveolar processes, and bone tissues that support them (22). As a consequence, maxillary cannot be ignored at the time of the inferior incisor positioning, within the dental arch (23).

Betzenberger et al. (24), in their paper, report that Hasund y Böe modified the Steiner analysis, and developed an equation of multiple regression for the position of the inferior incisors using the ANB, and two skeletal measures as guide variables (11). Schulhof et al. (25) report a study realized by Linder- Aronson on 60 patients, observed that there is a significant correlation between inclination of the lower incisor and the ANB angle, having therefore, a clear relation between maxillary bone base and the inclination of lower incisor. Given the variability of the ANB angle, during determination of the skeletal malocclusion, caused by rotation of the maxilla and mandible, and due to the positioning variable of the Nasion point, we have preferred, on the contrary, to take as defining measurement, the Wits appraisal of the skeletal malocclusion.

Solow et al. (26) through numerous studies, arrived to the conclusion that the upper incisor was directly related with length and the maxillary prognathism. Equally, a mandibular prognathism is compensated at the level of the superior incisor through its proinclination. Moreover, front an increase in length of the maxillary, or its prognathism, the inferior incisor proinclines in order to compensate this situation. He confirms a relation statistically significant between incisor inclination and the maxillo-mandibular relationship at sagittal level using correlation analysis. In our study we observed a higerproinclination in Class II, more accused in dolicofacial patterns; while the position is more retroinclinated in Class III, overcoat in braquifacial patterns.

Tweed (27) established the importance of the relation between the inclination of the inferior incisor and the mandibular plane, establishing between them a determined angular measure. In our study, we found similar results. It was observed a relation statistically significant between the inclination of the lower incisor and the mandibular plane in the different malocclusions.

Tweed (27) reports in his article how Downs stated using, at the beginning, the plane A- Pg, to evaluate the position of the lower incisor, and the modifications that it has to be done during the orthodontic treatment. Opposite to Downs’ results, in our study the A-Pg plane is not one of the reference lines to provide data more valuables when analyzing the position and inclination of the lower incisor because it does not produce results statistically significant, but this plane results statistically significant when we analyzed the facial patterns.

The anterior cranial base, measures from Sella to Nasion, is a parameter that perfectly describes the compensation realized by the lower incisor malocclusion. Horowitz y Hixon, as report Hasund and Ulstein in their paper (28), suggested that a correlation coefficient greater than 0.8 indicates a biologically significant entailment for a clinical prediction. Same correlation factor was found by Handelman (11) between the anterior cranial base and the lower incisor. These results agree with our studies at the level of skeletal malocclusions, and respect to the inherent differences to the facial pattern.

However, the interincisive angle results, according the results obtained in our study, and according other studies previously made, do not present a correlation substantially significant with the skeletal variations, both vertical and sagittal (28).

As pointed Schudy (29), the lower incisor inclination is closely related with the occlusal plane. However, in this study we have found differences in Class II dolicowith all the facial patterns of Class III; and Class I braqui; and between Class III dolico with all Class II paterns and with Class I meso and dolicofacial.

## Conclusions 

1. In Class II there are a lower incisor proinclination, higher in dolicofacial patterns, basedonthe analysis of the anterior cranial base, IMPA, Frankfurt plane, McHorris analysis and oclusal plane.

2. In Class III is produced a lower incisor retroinclination, more evident in braquifacial patterns, basedonthe analysis of the anterior cranial base, IMPA, and oclusal plane.

3. The McHorris analysis, and the inferior incisor inclination with respect to the anterior cranial base, the Frankfurt plane, and the occlusal plane, respectively, all maintain a directly proportionalrelation.

4. Finally, the inclination of the inferior incisor respect to the mandibular plane, the analysis outlined above, as well as the Holdaway and Ricketts analyses, all exhibit a relation which is inversely proportional.

5. In future studies, we will expand the sample, which could increase the methodology and support our results.

## References

[1] Harvold EP (1968 ). The role of function in the etiology and treatment of malocclusion. Am J Orthod.

[2] Corelius M, Linder-Aronson S (1976). The relationship between incisor inclination and various reference lines. Angle Orthod.

[3] Bibby RE (1980). Incisor relationship in different skeletofacial patterns. Angle Orthod.

[4] Bjork A (1963 ). Variations in the growth pattern of the human mandible: longitudinal radiographic study by the implant method. J Dent Res.

[5] Enlow DH, Kuroda T, Lewis AB (1971). Intrinsic craniofacial compensations. Angle Orthod.

[6] Sinclair PM, Little RM (1985 ). Dentofacial maturation of untreated normals. Am J Orthod.

[7] Casko JS, Shepherd WB (1984). Dental and skeletal variation within the range of normal. Angle Orthod.

[8] Kim JY, Lee SJ, Kim TW, Nahm DS, Chang YI (2005). Classification of the skeletal variation in normal occlusion. Angle Orthod.

[9] Ishikawa H, Nakamura S, Iwasaki H, Kitazawa S, Tsudaka H, Sato Y (1999). Dentoalveolar compensation related to variations in sagittal jaw relationships. Angle Orthod.

[10] Knösel M, Attin R, Kubein-Meesenburg D, Sadat-Khonsari R (2007). Cephalometric assessment of the axial inclination of upper and lower incisors in relation to the third-order angle. J Orofac Orthop.

[11] Handelman CS (1996). The anterior alveolus: its importance in limiting orthodontic treatment and its influence on the occurrence of iatrogenic sequelae. Angle Orthod.

[12] Turvey T, Hall DJ, Fish LC, Epker BN (1982 ). Surgical-orthodontic treatment planning for simultaneous mobilization of the maxilla and mandible in the correction of dentofacial deformities. Oral Surg Oral Med Oral Pathol.

[13] Solow B (1980). The dentoalveolar compensatory mechanism: background and clinical implications. Br J Orthod.

[14] Takada K, Petdachai S, Sakuda M (1993). Changes in dentofacial morphology in skeletal Class III children treated by a modified maxillary protraction headgear and a chin cup: a longitudinal cephalometric appraisal. Eur J Orthod.

[15] Worms FW, Isaacson RJ, Speidel TM (1976). Surgical orthodontic treatment planning: profile analysis and mandibular surgery. Angle Orthod.

[16] Aki T, Nanda RS, Currier GF, Nanda SK (1994). Assessment of symphysis morphology as a predictor of the direction of mandibular growth. Am J Orthod Dentofacial Orthop.

[17] Yamada C, Kitai N, Kakimoto N, Murakami S, Furukawa S, Takada K (2007). Spatial relationships between the mandibular central incisor and associated alveolar bone in adults with mandibular prognathism. Angle Orthod.

[18] Jacobson A (1975 ). The "Wits" appraisal of jaw disharmony. Am J Orthod.

[19] Biradar AK, Madanagowda SB (2010). Establishment of South Indian soft tissue cephalometric norms using profile angles and esthetic analysis. World J Orthod.

[20] Lahlou K, Bahoum A, Makhoukhi MB, Aalloula H (2010). Comparison of dentoalveolar protrusion values in Moroccans and other populations. Eur J Orthod.

[21] Ioi H, Nakata S, Nakasima A, Counts AL (2007). Comparison of cephalometric norms between Japanese and Caucasian adults in antero-posterior and vertical dimension. Eur J Orthod.

[22] Knösel M, Jung K (2011). On the relevance of "ideal" occlusion concepts for incisor inclination target definition. Am J Orthod Dentofacial Orthop.

[23] Knösel M, Engelke W, Attin R, Kubein-Meesenburg D, Sadat-Khonsari R, Gripp-Rudolph L (2008). A method for defining targets in contemporary incisor inclination correction. Eur J Orthod.

[24] Betzenberger D, Ruf S, Pancherz H (1999). The compensatory mechanism in high-angle malocclusions: A comparison of subjects in the mixed and permanent dentition. Angle Orthod.

[25] Schulhof RJ, Allen RW, Walters RD, Dreskin M (1977). The Mandibular Dental Arch: part I, lower incisor position. Angle Orthod.

[26] Solow B, Tallgren A (1977). Dentoalveolar morphology in relation to craniocervical posture. Angle Orthod.

[27] Tweed CH (1946 ). The Frankfort-mandibular plane angle in orthodontic diagnosis, classification, treatment planning, and prognosis. Am J Orthod Oral Surg.

[28] Hasund A, Ulstein G (1970 ). The position of the incisors in relation to the lines NA and NB in different facial types. Am J Orthod.

[29] Schudy ff (1965 ). The rotation of the mandible resulting from growth: Its Implications in orthodontic treatment. Angle Orthod.

